# Manual of Midwifery

**Published:** 1897-03

**Authors:** 


**Manual of Midwifery.** By W. E. Fothergill, M.B. Pp. xviii., 484. Edinburgh: William F. Clay. 1896.

Dr. Fothergill has produced a very useful and readable volume, which embodies more especially the teaching of the Edinburgh school. The chapters on the phenomena and diagnosis of pregnancy and the development of the early ovum are especially noteworthy; that on the mechanism of delivery is a very clear exposition of the different theories which are advanced to explain the phenomena occurring during the progress of delivery, and the points for and against their correctness are discussed in a lucid and able manner. The summaries given at the end of each section clearly and briefly set forth the chief points to be remembered, and very materially enhance the value of the work as a manual for students. The treatment of puerperal fever might have been extended with advantage, considering the enormous importance of the subject; treatment by curetting is mentioned, but only as regards its possible dangers, while the *technique* of the operation on the correct carrying out of which its success depends is entirely omitted. To state that "the most recent method of removing the source of infection is to remove the whole uterus by hysterectomy," without giving the special indications for performing an operation of such gravity, can only be regarded as a very inadequate and loose description of this method. No one, we hope, would recommend this method of treatment for cases of sapræmia, for example, where the removal of effete matter from the uterine cavity would probably be all that was necessary; this, however, is the impression left on one's mind by reading the paragraph.

The author states as regarding the puerperal period that "bed should be kept for 10 days": this again is a statement that is certainly true, as ten days should be looked upon as the minimum; but the omission of the reasons for this, *e.g.* the rate of subsidence of the fundus and the average time at which it ceases to be an abdominal organ, with the various reasons which prolong this time, is a serious fault. This particular subject is one on which hints to the student or young practitioner are most valuable in securing the prophylactic treatment of many local disorders arising from the empirical management of the puerperal state. It is above all things important that the necessity of prolonging the lying-in period under certain conditions should be laid down distinctly, together with the conditions which call for it. No allusion is made to methods of preparing, for the use of the infant, cow's milk so treated as to render its composition nearly that of human milk (the so-called humanised milk), nor to the treatment of vomiting in the nurseling. The points just mentioned—the management of the puerperal state, the feeding of the infant, and the treatment of its earliest disorders—are just those which give the greatest trouble to the young practitioner, and a full description of these would greatly enhance the value of the work.

Several conditions producing risk to the mother and child are not sufficiently dealt with; for example, the only treatment of accidental hemorrhage given is rupture of the membranes, followed by version or forceps as soon as dilatation is sufficiently advanced, while hysterectomy is mentioned as an alternative in some cases. This is not an adequate description, for the opinions of the Rotunda school, Robert Barnes, and others should have been mentioned, even if the author considers his own recommendations superior. Prolapse of the funis, too, should have received fuller treatment; no single method is applicable to all cases, and the various conditions which call for different methods of treatment in any case of complication should be given.

The theoretical part of the subject is excellent, but no work of the kind can suffer from excess of detail and elaboration in the practical part of the subject.

